# Supplementary material for: 3D cell aggregates amplify diffusion signals
Source: PLoS One. 2024 Sep 12;19(9):e0310109. doi: 10.1371/journal.pone.0310109 (PMC12139657; doi:10.1371/journal.pone.0310109)
Supplement: S1 Table — (ZIP) [file pone.0310109.s001.zip › S1_Table.pdf]

Supplementary Table S1

Spheroids were transferred to a 96-well flat bottom ultra-low attachment plate (Corning) and medium was changed to 75 or 100 μL (8 replicates per volume) HepaRG medium with 11.1 mM glucose and 870 nM insulin. Medium samples were taken after 1, 5, and 10 min and HepaRG medium with 11 mM glucose and 870 nM insulin was sampled as 0 min control. For samples where starting volume was 100 μL, a medium sample was also taken after 4 h. For samples with starting volume of 75 μL, all medium was removed after 10 min sampling and 100 μL new medium was added. From these incubations, medium was sampled after 19 h. The measured data is provided in Supplementary Tables S1-S3.

Supplementary Table S1: Measured glucose concentration of medium before adding the spheroids

11.34	11.15	10.49	11.79	11.21	10.95	11.26	10.76
