# Supplementary material for: 3D cell aggregates amplify diffusion signals
Source: PLoS One. 2024 Sep 12;19(9):e0310109. doi: 10.1371/journal.pone.0310109 (PMC12139657; doi:10.1371/journal.pone.0310109)
Supplement: S2 Table — (ZIP) [file pone.0310109.s002.zip › S2_Table.pdf]

Supplementary Table S2

Supplementary Table S2: Measured Glucose concentration at time points 1, 5, 10 min, and 4h in 100 μL incubations.

Replicate number	1	2	3	4	5	6	7	8
**1min**	9.94	11.05	10.95	10.85	11.32	10.91	10.74	10.33
**5min**	12.09	11.03	11.35	11.46	10.92	10.92	11.31	10.43
**10min**	10.45	10.09	10.15	9.82	9.89	9.53	10.62	9.91
**4h**	10.24	10.00	NV [a]	9.72	9.77	9.75	9.25	8.43

[a] No value.
