# Supplementary material for: 3D cell aggregates amplify diffusion signals
Source: PLoS One. 2024 Sep 12;19(9):e0310109. doi: 10.1371/journal.pone.0310109 (PMC12139657; doi:10.1371/journal.pone.0310109)
Supplement: S3 Table — (ZIP) [file pone.0310109.s003.zip › S3_Table.pdf]

Supplementary Table S3

Supplementary Table S3: Measured Glucose concentration (GC) at times 1, 5, 10 min, and 19h in 75 μL incubations.

Replicate number	1	2	3	4	5	6	7	8
**1min**	9.96	11.00	9.89	10.64	10.58	9.67	10.96	10.93
**5min**	12.12	11.19	10.68	11.27	11.51	11.23	11.07	9.83
**10min**	10.71	10.69	11.68	11.15	11.04	10.97	11.31	10.61
**19h**	6.50	5.41	4.72	4.84	5.19	5.31	4.87	5.40
