# Supplementary figures and images for: 3D cell aggregates amplify diffusion signals
Source: PLoS One. 2024 Sep 12;19(9):e0310109. doi: 10.1371/journal.pone.0310109 (PMC12139657; doi:10.1371/journal.pone.0310109)

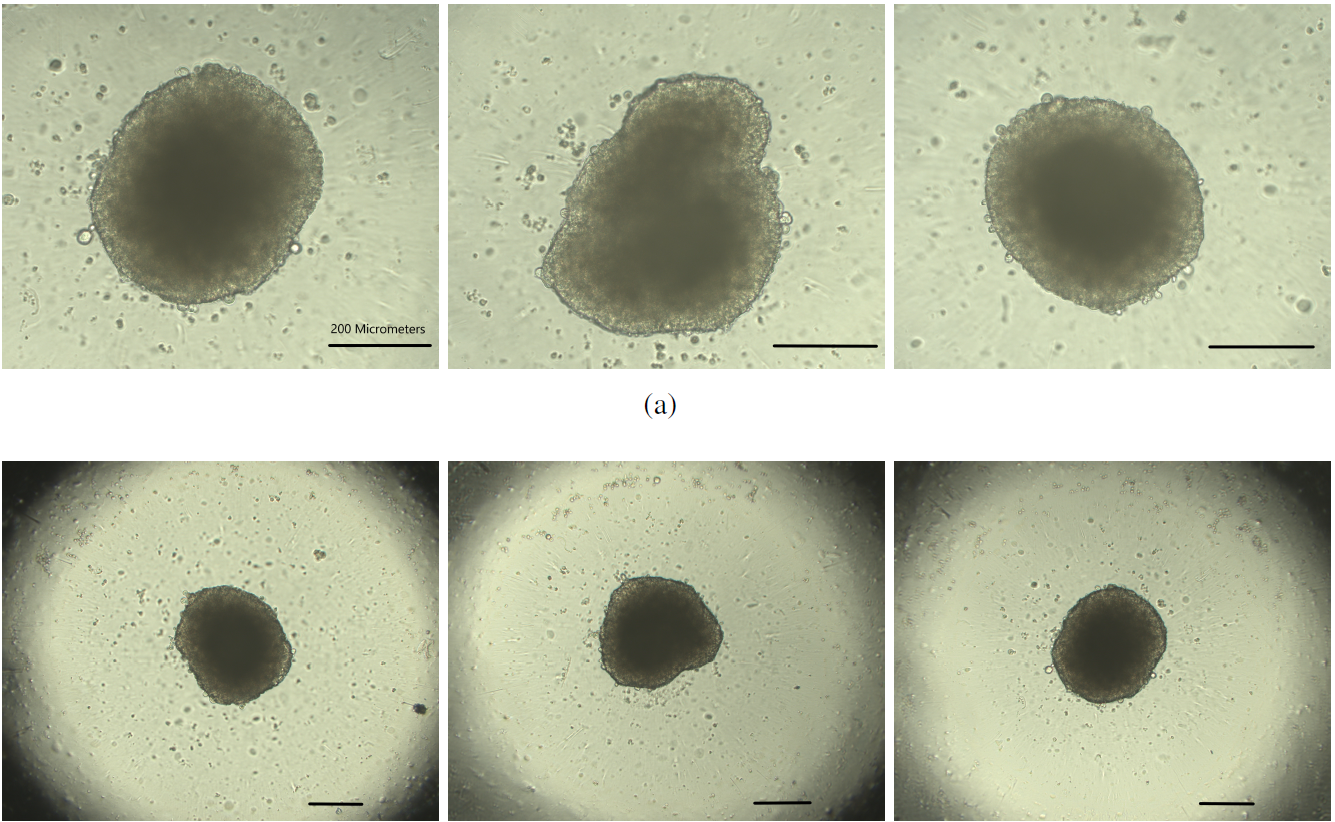

Supplement: S1 Fig — (a) Microscopic images of three liver spheroids at 10X magnification (scale bar is 200 μm). (b) Microscopic images of three spheroids at 4X magnification (scale bar is 200 μm). (ZIP) [file pone.0310109.s004.zip › S1_Fig.png]

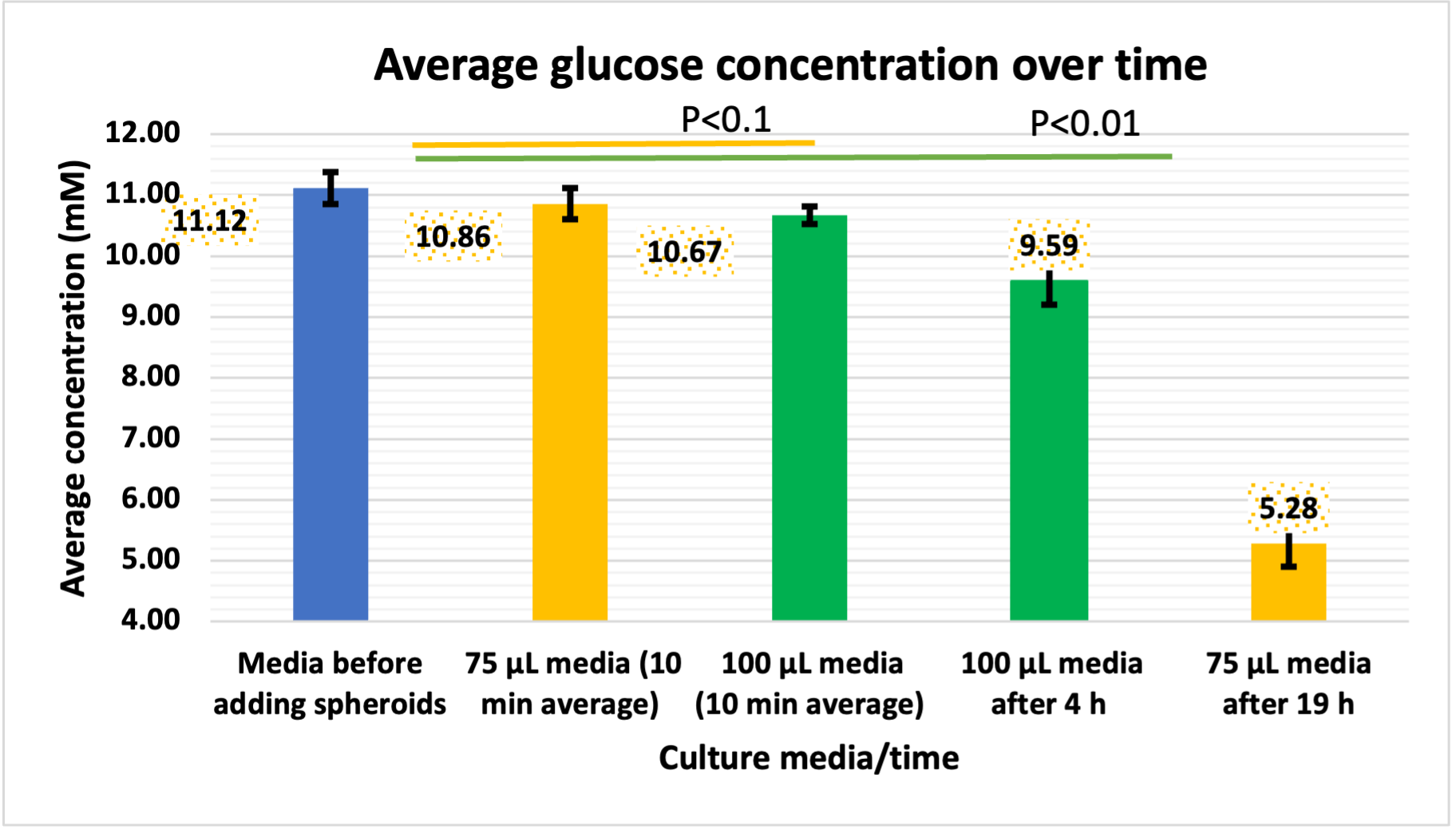

Supplement: S2 Fig — (ZIP) [file pone.0310109.s005.zip › S2_Fig.png]
