# Supplementary material for: 3D cell aggregates amplify diffusion signals
Source: PLoS One. 2024 Sep 12;19(9):e0310109. doi: 10.1371/journal.pone.0310109 (PMC12139657; doi:10.1371/journal.pone.0310109)
Supplement: S2 Appendix — (ZIP) [file pone.0310109.s007.zip › S2_Appendix.pdf]

Appendix S2

The following Matlab code implements the PBS to generate Fig.2.

System parameters

```
N=floor(1000000);%Initial number of molecules in the medium
Nc=25000;%Number of cells in one spheroid
vc=1.7e-15;%
R1=226e-6;% Spheroid radius
eps1=1-(Nc*vc)/(4*pi/3*R1^3);% \epsilon: spheroid (volume) porosity
D1=1e-9;%Assumed diffusion coefficient for molecules inside the medium
D2=eps.^1.5*D1;%Effective diffusion coefficient inside the spheroids
kapa=sqrt(D1./D2);
eps=eps1^(2/3);%surface porosity
Ro=3.2e-3;%Well radius
Ho=2.4e-3;%Well height
```

Placing Spheroids at the bottom of the well

```
Nsph=40;% number of spheroids
[XY2,XYi2,XYZ_sph]=XYZ_sph1(Nsph,Ro,R1);% Defined function
```

Initialization of molecule locations in the medium

```

Pc1=[];% storing the molecules positions in medium in each time slot
Pc2=[];%storing the molecules positions inside the spheroids in each time slot

Pc1(:,1:2)=2*Ro*rand(N,2)-Ro;
Pc1(:,3)=Ho*rand(N,1);
[k,dist] = dsearchn(XYZ_sph,Pc1);
ch_row1=find(dist>R1);
Pc1=Pc1(ch_row1,:);
dis_reci=vecnorm((Pc1(:,1:2))');
ch_row2=find(dis_reci<Ro);
Pc1=Pc1(ch_row2,:);
```

Initialization of molecule locations inside spheroids

```
N2=floor(N*kapa*2.8/11);%Initial number of molecules inside the spheroids
Pc2(:,1:2)=2*Ro*rand(N2,2)-Ro;
Pc2(:,3)=Ho*rand(N2,1);
[k2,dist2] = dsearchn(XYZ_sph,Pc2);
ch_row12=find(dist2<=(R1));
Pc2=Pc2(ch_row12,:);
Ind2=k2(ch_row12);
```

```
N=length(ch_row2);
V1=(pi*Ro^2*Ho-Nsph*4/3*pi*R1^3);%volume of liquid in the well
V2=Nsph*4/3*pi*R1^3;%volume of spheroid
Cout=N/V1;
```

Diffusion of molecules in the system

```
T(1)=0;
dt=1e-1;

for k=1:30000
    if mod(k,500)==0
        k
    end
    T(k+1)=T(k)+dt;
    dPc1=(sqrt(2*D1*dt))*randn(size(Pc1,1),3);
    dPc2=(sqrt(2*D2*dt))*randn(size(Pc2,1),size(Pc2,2));
    Pc2=Pc2+dPc2;
    Pc1=Pc1+dPc1;
```

Molecules hitting well boundaries and top

```
    %from side face
    dis_reco=vecnorm((Pc1(:,1:2))'/Ro);
    ao=(dis_reco>1);%+(dis_rec2<rR);
    ch_rowo=find(ao);
    Pc1(ch_rowo,:)=Pc1(ch_rowo,:)-dPc1(ch_rowo,:);
    %from top and bottom
    ao=(0<Pc1(:,3)& Pc1(:,3)<Ho);%+(dis_rec2<rR);
    ch_rowo=find(~ao);
    Pc1(ch_rowo,:)=Pc1(ch_rowo,:)-dPc1(ch_rowo,:);
    %Divide Pc1 to bottom and top molecules
    aD=(Pc1(:,3)<2*R1);
    ch_rowD=find(aD);
    noch_rowD=find(~aD);
    Pc1D=Pc1(ch_rowD,:);
```

```
dPc1D=dPc1(ch_rowD,:);

Pc1T=Pc1(noch_rowD,:);%Pc1 in other parts
dPc1T=dPc1(noch_rowD,:);
```

Molecules in the medium hitting spheroids boundaries

```
[Ind,dist] = dsearchn(XYZ_sph,Pc1D);
a=(dist<R1);
a_pr=rand(size(a));
a=(dist<R1).*(a_pr<eps);% with window at boundary
noch_row=find(~a);
ch_row=find(a);
a_back=(dist<R1).*(a_pr>=eps);
ch_row_back=find(a_back);

if size(ch_row,2)*size(ch_row,1)>=1
    Pc1D(ch_row,:)=Pc1D(ch_row,:)-dPc1D(ch_row,:);


    A=(vecnorm(Pc1D(ch_row,:)'-XYZ_sph(Ind(ch_row),:)')-R1)./...
    (vecnorm(Pc1D(ch_row,:)'-XYZ_sph(Ind(ch_row),:)')-...
    vecnorm(Pc1D(ch_row,:)'
    +dPc1D(ch_row,:)'-XYZ_sph(Ind(ch_row),:)'));

    dPc1D(ch_row,:)=dPc1D(ch_row,:).*(A'+(1-A')*sqrt(D2/D1));
    Pc2=[Pc2;Pc1D(ch_row,:)+dPc1D(ch_row,:)];
    dPc2=[dPc2;dPc1D(ch_row,:)-dPc1D(ch_row,:)];
    Ind2=[Ind2;Ind(ch_row)];
end
Pc1D(ch_row_back,:)=Pc1D(ch_row_back,:)-dPc1D(ch_row_back,:);
Pc1=[Pc1T;Pc1D(noch_row,:)];
```

Molecules insode spheroids hitting the spheroids boundaries

```
if size(Pc2,1)>0


    dis_rec2=vecnorm((Pc2(:,:)-XYZ_sph(Ind2,:))');
    a2=(dis_rec2>R1);
    a2_pr=rand(size(a2));
```

```
        a2=(dis_rec2>R1).*(a2_pr<eps);% with window at boundary
        noch_row2=find(~a2);
        ch_row2=find(a2);
        a2_back=(dis_rec2>R1).*(a2_pr>=eps);
        ch_row_back2=find(a2_back);
        Pc2(ch_row_back2,:)=Pc2(ch_row_back2,:)-dPc2(ch_row_back2,:);
        if size(ch_row2,2)*size(ch_row2,1)>=1
            Pc2(ch_row2,:)=Pc2(ch_row2,:)-dPc2(ch_row2,:);
            A=(vecnorm(Pc2(ch_row2,:)'+dPc2(ch_row2,:)'-...
            XYZ_sph(Ind2(ch_row2),:)')-
            R1)./(vecnorm(Pc2(ch_row2,:)'+dPc2(ch_row2,:)'-...
            XYZ_sph(Ind2(ch_row2),:)')-
            (vecnorm(Pc2(ch_row2,:)'-XYZ_sph(Ind2(ch_row2),:)')));
            dPc2(ch_row2,:)=dPc2(ch_row2,:).*((1-A')+A'*sqrt(D1/D2));
            Pc2(ch_row2,:)=Pc2(ch_row2,:)+dPc2(ch_row2,:);

            Pc1=[Pc1;Pc2(ch_row2,:)];
            Pc2=Pc2(noch_row2,:);
            Ind2=Ind2(noch_row2,:);
        end
```

```
        N_Rec1(k)= size(Pc1,1);% total number of molecules in medium
        N_Rec2(k)= size(Pc2,1);% total number of molecules inside the spheroids



    end
    end

hold on
plot(T(2:end),N_Rec1/V1/Cout)
plot(T(2:end),N_Rec2/V2/Cout)
```

Meshing the well bottom and random positioning of the spheroids

```
function [XY2,XYi2,XYZ_sph]=XYZ_sph1(Nsph,Ro1,R1)%XY2 gives the center
%of spheroids, and XYi2 their indices
Ro=Ro1-1*R1;% to consider the radius of spheroids at the boundaries of well
N=Nsph*4*Ro^2/(pi*Ro^2);
NX=floor(sqrt(N));
DelX=2*Ro/NX;
X=[-Ro+DelX/2:DelX:Ro]';
NY=floor(sqrt(N));
DelY=2*Ro/NY;
Y=[-Ro+DelY/2:DelY:Ro]';
[X1,Y1]=meshgrid(X,Y);
```

```
X1=reshape(X1,[NX*NY,1]);
Y1=reshape(Y1,[NX*NY,1]);
XY=[X1,Y1];
R=sqrt(X1.^2+Y1.^2);
f_row=find(R<Ro1);
XY1=XY(f_row,:);
Ran=randperm(length(XY1));
XY1=XY1(Ran,:);
XY2=XY1(1:Nsph,:);
Xi=[1:NX];
Yi=[1:NY];
[Xi1,Yi1]=meshgrid(Xi,Yi);
Xi1=reshape(Xi1,[NX*NY,1]);
Yi1=reshape(Yi1,[NX*NY,1]);
XYi=[Xi1,Yi1];
XYi1=XYi(f_row,:);
XYi1=XYi1(Ran,:);
XYi2=XYi1(1:Nsph,:);
XYZ_sph=[XY2,zeros(Nsph,1)+R1];
```
