# Supplementary material for: 3D cell aggregates amplify diffusion signals
Source: PLoS One. 2024 Sep 12;19(9):e0310109. doi: 10.1371/journal.pone.0310109 (PMC12139657; doi:10.1371/journal.pone.0310109)
Supplement: Attachment — Submitted filename: Response to Reviewers.pdf [file pone.0310109.s008.pdf]

Response to the Reviewer's Comments for the Manuscript: '3D Cell Aggregates Amplify Diffusion Signals'

We would like to thank the Editor and reviewers for their helpful comments and valuable suggestions for our manuscript. Also, we would like to thank the Editor for his timely processing of our manuscript. In the following, we provide a detailed point–to–point response to all the reviewers' comments and explain how these comments have been accounted for in the revised manuscript. The reviewers' comments and our responses are in *italic* and regular fonts, respectively. Text that has been added or modified in response to the reviewers' comments is highlighted in blue , and deleted text is highlighted in red in the revised manuscript to facilitate the review process. The reference numbers in our responses correspond to the revised manuscript.

Response to Reviewer 1

Overall Comment: *This paper is about diffusion signal amplification by 3D cell cultures. The topic tries to answer to the interesting question and this could draw attention from broad field of research.*

Response: Thank you for your supportive comment.

Comment 1: *However the presented results are not convincing enough to support the author's claims on the signal amplification. This work summarizes preliminary results but to be published in any journal, I believe that more clear results must be provided. So I suggest that authors can make more measurements results and through statistical study.*

Response: Thank you for your comment. From an experimental perspective, we acknowledge the reviewer's suggestion that this work provides preliminary results and can benefit from additional measurements and experiments. However, our manuscript supports the concept of diffusion signal amplification in several ways, laying the groundwork for future research and experimentation across diverse cell types and environments. Firstly, in proposition 1, we have mathematically proved the amplification factor. Also, we have proposed a particle-based simulator that replicates the Brownian motion of individual molecules and enables tracking of molecules within the environment. The PBS confirms the amplification factor and aids in the design of future experiments. Further, our experiments using Human liver spheroids and a glucose assay agree with the proposed theory and derived properties.

Additionally, in the revised manuscript, we have added and modified several paragraphs in the Theoretical and Experimental Methods sections as well as in the Results section to clarify the above arguments. These changes are highlighted in blue.

Comment 2: *The graphs must be provided with higher quality of image with errors bars as well.*

Response: Thank you for your comment. We have added error bars in the presentation of experimental results where we have multiple replicates (Please see Fig. 6). However, PBS conceptually involves averaging the behaviours across numerous molecules. Moreover, our PBS uses a simplified environment avoiding the experimental noises and uncertainties that lead to different results for replicates. Therefore, the observed concentration represents the mean value with minimal (negligible) variance to be depicted by error bars.

We have also enhanced the quality of the figures, specifically by improving the clarity of the legends, axis titles, and values.

Comment 3: *I also suggest that authors should show microscopic photograph to show the cell cultures in 3D. They must also be linked to each measured results.*

Response: Thank you for your suggestion. We have included microscopic images of a couple of individual spheroids in the supplementary file. Each sample consists of a pool of 40 different spheroids; hence a measurement cannot be linked to a specific spheroid. Measurements are made from the medium before and after incubations with the spheroids and are not detected from any images. The spheroids are very uniform in size and shape and any variations would be averaged using 40 spheroids/sample.

Response to Reviewer 2

Overall Comment: *The present manuscript addresses a very important theme in biology, in which the scientist attempts to optimize human models for in vitro studies. 3D human cellular models are becoming the paradigm for drug screening and fundamental research since they are more realistic than animals and bypass the experimental animals' ethics. Although a huge progress has been made in few years, as any emerging field in biology, there are several open questions and bottlenecks. The present manuscript focus in one of these, which is related to prediction models for signals diffusion and how to measure their accuracy. The paper brings information on the development of a reliable biophysical model for the 3D cellular aggregates. More specifically on models for mass diffusion, which is critical for optimization and simulation of physiological aspects concerning drug discovery or even fundamental science.*

The information provided on the manuscript is based in pilot experiments and it would be beneficial if authors could improve the sample number to consider a more reliable dataset. However, the data seems to be consistent and reproducible, so I do not have restrictions. Experimentally, the authors used a well stablish protocol for creating liver organoids and compare the results with simulated models. Also, they are one of few papers addressing the "3DCA border" in the models and making clear the importance in the mathematical model. Overall the text is well written in good English, but I raise few minor points as suggestions:

Response: Thank you sincerely for your supportive perspective and invaluable comments. We believe that your comments have significantly enhanced the quality of our revised manuscript.

Minor Comments:

Comment 1: *Although the introduction is very complete and may be beneficial for a non-expert reader, it should be shorted, referring to already published papers, especially in concerning the importance of 3DCA.*

Response: Thank you for your comment. In the revised manuscript, we have shortened the Introduction by removing the second paragraph which discussed the importance of biophysical models in general and accompanying references.

Comment 2: *It should be clearer that authors are assuming a homogeneous extracellular space within the 3DCA. A conventional (2D) histology reveals it is not. But how it will impact the model is not clear for me and it deserves some discussion. Maybe consider a high-resolution X-ray tomography for 3D quantification of the inhomogeneity.*

Response: Thank you for your constructive comment. A homogenized environment importantly implies that the diffusion coefficient is the same in the entire environment. We have assumed this for the simplicity of the proof for Proposition 1. However, the provided boundary condition in Proposition 1 concerns the diffusion coefficients very close to the boundary where the diffusion coefficient could be assumed fixed. Irrespective to the real experiment, the PBS also makes a homogeneous assumption as an independent approach to evaluate the proposition using this assumption. We have added the following sentence after Proposition 1 for clarification:

"For simplicity of the presentation, we proposed Proposition 1 under the assumption of homogenous environments on both sides of the boundary. However, the proof holds for non-homogenous environments where the diffusion coefficient is a function of location. In such an environment, the diffusion coefficient variability across space can be managed by adjusting the time interval Δt, thus controlling the proximity to the border where the diffusion coefficient can be reasonably approximated as fixed."

Regarding the PBS, we agree that this assumption does not accurately simulate the experiment environment. As the reviewer rightly mentions, from an experimental perspective, the diffusion coefficient varies from the boundary towards the core of the 3DCA (e.g., a spheroid). However, we have justified this by including the following part in the "Particle-based simulator" subsection:

"It is noteworthy that the diffusion coefficient within a spheroid may vary according to the local cell size and density (e.g., it may be lower in a necrotic core with dead and dying cells). However, using the simplified homogenous model in PBS is justified due to the following reasons:

(I) The PBS uses an average effective diffusion coefficient for inside the 3DCA that mitigates the impact of variation. (ii) We focus on the initial 10 minutes when the molecules may not get very far from the boundary in the real experiment, particularly considering the smaller diffusion coefficient compared to the outside. The region of the spheroid near the surface is more likely to be uniform and have a comparable diffusion coefficient. (iii) Further, the protocol used to form the liver spheroids is based on [2] where the authors demonstrate the viability of the cells at the core. Thus, we expect that there is no necrotic region present. "

Comment 3: *In the "Results" section, there are missing some discussions of the presented data. For instance, in Fig. 4, 5 and 6.*

Response: Thank you for your comment. The key focus of Figure 4 lies in illustrating the variations of both k and ϵ in terms of the number of cells N_c. This information has been provided in the manuscript. We have made slight revisions to the sentences for completeness, as follows:

"As observed in Fig. 4, k increases exponentially and ϵ demonstrates a linear decrease with an increase in N_c. "

Regarding Fig. 5, it primarily serves to visualize the data used to generate the results depicted in Figs. 6 and 7, which we have extensively discussed in accordance with the hypotheses in the manuscript. We have explicitly mentioned this point in the Results section by adding the following sentence:

"Fig. 5 visualizes the data used to generate the results depicted in Figs. 6 and 7."

Regarding Fig. 6, we have comprehensively discussed the noticeable decrease in glucose concentration within the first 10 minutes across both 75 and 100 μL experiments confirming the theoretical results. Further, we have conducted the hypothesis test to compare the mean glucose concentrations before and after adding the spheroids, elaborating on this in the original manuscript.

Comment 4: *In line 426-432, the information of radius and volume was already presented in methodology and should be omitted.*

Response: Thank you for your comment. It is corrected in the revised manuscript.

Comment 5: *Please review lines 429-432. The information "... we have* $\epsilon = 0.1$ *and 430 correspondingly* $k = 4.49\ldots$*" is not presented in Figure 4, which shows the porosity and k for 25000 cells as below 0.1 and more than 5.5 , respectively.*

Response: Thank you for your comment. We have revised the figure by incorporating the correct cell volume of 1.7×10^{-5}. In the updated manuscript, we now report $k = 4.87$ and $\epsilon = 0.12$. We note that the provided PBS utilized the correct value of cell volume in the original manuscript.

Comment 6: *In the legend of Figure 6 should considering a first sentence as title.*

Response: Thank you for your comment. We have added the following title to the caption of Fig. 6:

"Mean GC during 10 minutes after introducing the spheroid to the medium over 8 replicates of the 100 and 75 μL incubations. "

Comment 7: *In Figure 6 consider including "glucose" in the plots' titles (e.g. "Average glucose concentration...")*

Response: Thank you for your comment. It is changed in the revised manuscript.

Comment 8: *Lines 452-454 are almost identical to lines 447-449. Please correct by merging both information.*

Response: Thank you for your comment. It is corrected in the revised manuscript.

Comment 9: *Lines 465-468 brings introduction in statistical hypothesis test. It is not necessary and can be omitted. But, if author intend to keep, please move to methodology.*

Response: Thank you for your comment. We have removed it in the revised manuscript.

Concluding Comment *Considering the presented above and how complex and expensive is a 3D human cellular model, I think any improvement and optimization is beneficial. Then, my opinion is favorable for the publication of this manuscript.*

Response: Thank you sincerely for your supportive perspective and invaluable comments. We believe that your comments have significantly enhanced the quality of our revised manuscript.

Response to Reviewer 3

Overall Comment: *Summary: In this paper, the authors explore the diffusive transport of glucose between 3D cell aggregates and the surrounding medium and focus on the influence of the boundary. Mathematically, the authors model diffusion in the medium as free diffusion, and diffusion in the 3D cell aggregate using free diffusion with an effective diffusion coefficient that assumes that transport in the 3D cell aggregate only occurs in the extracellular fluid. The authors also consider a discrete model of Brownian motion with different rates of movement for particles in the media and in the 3D cell aggregate. Experimentally, the authors incubate liver spheroids in a low glucose condition and then transfer them to a high glucose condition and measure the glucose concentration (focusing on the first 10 minutes). The authors then relate the results of the mathematical model to the experimental results. Overall, I feel that the study is of potential interest to the community. However, I feel that key methodological details are missing (particularly with the particle-based model) that would help in interpreting the results and that the structure of the manuscript should be modified. Further, I feel that it would help to clarify how K can be estimated from data and how the results can be extended to other situations. Additional detailed comments are included below.*

Response: Thank you for your positive perspective and supportive comments. We have carefully addressed your comments and concerns which have improved the manuscript.

Major comments:

Comment 1.1: *Key mathematical modelling details are not clear. In particular, it is not clear how to reproduce the particle-based simulator. - Line 306 – How is k=4.49 obtained? It looks like the authors use the diffusivities from Line 330 and Eq 5. If so, this should be made clear at Line 306.*

Response: Thank you for your comment. The reviewer's ambiguity regarding PBS arises from

our calculation of the k values where we explain the PBS, which might imply that we utilize these values in the simulator. However, it's crucial to clarify that the simulator operates independently of the k value. It only relies on diffusion coefficients and random molecular steps, enabling us to evaluate the k value as obtained in Proposition 1. To prevent any confusion, we have removed the k value calculations when explaining PBS.

Comment 1.2: - *Particle-based simulator. Do the authors model the full 3D experimental well or do they model one 3D cellular aggregate? If it is multiple 3D cellular aggregates, are they placed uniformly throughout the domain? Are the 3D cellular aggregates assumed to be spherical, or are other geometries chosen to represent their geometry? More details would be helpful.*

Response: Thank you for your constructive comment. We have simulated the full 3D experimental well. We have meshed the well base and randomly selected 40 grids for spheroid placement to avoid overlap. We have assumed spherical spheroids. For clarification, we have included the following details in the subsection of partcile-based simulator:

"For our simulation, we considered a flat-bottomed well with a radius of $R_w = 3.2$ mm and a height of 6.2 mm. Forty spherically shaped spheroids with radius $R_s = 226$ μm were randomly distributed over the bottom of the well. "

In addition to the above changes, we have included more details in this section in response to other comments that clarify the PBS and its reproducibility.

Comment 2: *Structure of the manuscript. I am not sure that the heading "Experimental methods" accurately describes the content included in this section. "Rapid concentration reduction in culture medium" seems to be a mathematical modelling argument that would be better placed in the Theoretical methods section or the Results section. The particle-based simulator seems like it should be placed in the Theoretical methods section. Fig 2 and the associated discussion seems to better placed in the Results section.*

Response: Thank you for your comment. In the revised manuscript, we moved the subsection "Rapid concentration reduction in culture medium" to the theoretical section. However, we retained the particle-based simulator and the resulting Fig. 2 in the Experimental section, as it is used to

design the experiment.

Comment 3: *The mathematical proof in Appendix 1 would benefit from further justification on the points below. - Particles located in $x < -3sigma_0$ have a non-zero probability of crossing the boundary. These are excluded from the argument. Similarly, for particles on the right-hand side.*

- How is the probability determined? State which assumptions have been made. I think I know what these are from the description of the particle-based simulator, but this appendix should be self-contained for clarity.

- What is the definition of σ_s?

Response: Thank you for your clarifying comment. For clarification of the first two points, we have revised and extended the beginning of the proof as follows:

"A molecule located at left side region, $x < 0$, undergoes a Gaussian-distributed displacement with mean zero and a standard deviation of $\sigma_o = \sqrt{2D\Delta t}$, during a very short time interval Δt. During this time interval, the molecules within the range $x \in [-3\sigma_o, 0]$ have a not insignificant chance to traverse across the boundary to the right side. The molecules located at $x < -3\sigma_o$ have an exceedingly negligible chance, approximately 0.015% of moving beyond $3\sigma_o$ to the right side and overcoming the barrier within this time duration. "

We have also defined σ_s in the revised manuscript as $\sigma_s = \sqrt{2D_{\text{eff}}\Delta t}$ by adding a sentence.

Comment 4: *The literature review would benefit from examples of situations where one would expect jumps in the concentration of nutrient/oxygen/molecules at the media/3D cell aggregate interface and situations where one would not expect a jump in concentration. For example, it may be helpful to make comparisons to past experiments, e.g. Mueller-Klieser W, Freyer J, Sutherland R. Influence of glucose and oxygen supply conditions on the oxygenation of multicellular spheroids. British Journal of Cancer. 1986; 53:345–353.*

Response: Thank you for your comment. In the original manuscript, we have extensively covered several related works where the BC has been discussed or modelled. In particular, we have referenced [17] where the amplification factor is a possible explanation for the observed initial offset in glucose concentration reported. Further, we have reviewed works that define the amplification BC

as a unitary ratio, implying that the concentration is the same inside and outside the 3DCA border [8]-[11].

The impact of the amplification factor extends beyond transient behavior or local phenomena; it influences all 3D cellular aggregate (3DCA) experiments. This factor couples the diffusion equations both inside and outside 3DCAs, making it a fundamental property crucial for accurately building a biophysical model of the system.

In this work, we validated the amplification property through the proposed experiment. We have clarified the need to consider diverse cell types and varying experimental conditions as future directions as stated in the Conclusion Section:

"This was an initial pilot study using liver spheroids, and we require additional experimental data involving diverse cell types and varying conditions to more comprehensively characterize and capture the amplification property. The adoption of more precise measurement protocols and tools could prove invaluable in reducing the observed high variability."

Thus, given the general existence of the amplification property and the literature we have already cited to support it, we have not added the suggested paper to the literature review.

Comment 5.1: *I feel that the conclusions (Lines 501-515) overstate the importance of this result. -I agree that the result is of interest for 3D cellular aggregates surrounded by media over a very short duration. However, how do the results extend to typical experimental durations that range from hours to days where consumption is important? Further, how do the authors expect these results to extend to in vivo situations where 3D cellular aggregates are surrounded by other cells (where the difference in effective diffusivity would be smaller and k would more likely be approximately unity?)*

Response: Thank you for considering this point. The amplification factor characterizes the BC at the boundary. As mentioned in response to comment 1 of this reviewer, it inevitably affects the whole diffusion process at all times and throughout the whole environment resulting in an overall increase in molecular concentration within the 3DCA. In other words, the concentration profile of molecules consumed or remaining inside the 3DCA directly correlates with this factor as it couples the diffusion equations inside and outside the 3DCA. The amplification property remains

constant at the border, unaffected by the transient or equilibrium phases of diffusion. It constitutes a foundational characteristic of the 3DCA, independent of temporal variations or internal reactions within the 3DCA.

Our experimental design focuses on measuring concentration during the initial 10 mins, primarily to validate Proposition 1 and uncover the amplification property without constraining its influence in time or space. Within the first couple of minutes, we anticipate a lower impact from potential chemical reactions within the liver spheroid and reduced consumption of glucose molecules. Therefore, the molecule concentration change within the media will be dominated by the diffusion processes and boundary conditions and not the chemical reactions.

For clarification, we have added the following sentences to the subsection "Rapid concentration reduction in culture medium":

"However, it is essential to emphasize that the amplification factor remains constant and influences the diffusion process, regardless of whether reactions within the spheroid occur at faster or slower rates. "

Also, we have included the following part to the subsection Modeling of Diffusion BCs at the Border of 3DCA:

"It is important to highlight that while the amplification factor characterizes the BC at the border, it influences the whole diffusion process both inside and outside the 3DCA by coupling the diffusion equations of both sides through the corresponding BC. "

The proposed amplification factor is versatile and not limited to specific cases, suitable for modeling diffusion in both in vitro and in vivo settings. In scenarios where cell aggregates are surrounded by different types of neighbouring cells, the boundary conditions rely on the effective diffusion coefficients in both environments, determined by their respective porosities and tortuosities. This could result in an amplification factor of unity, or it may vary, being lower or higher than 1.

Comment 5.2: - *The amplification factor k appears to be calculated based on the diffusion coefficient in ref [2]. Line 511 – is the 'quantification of the amplification factor' referring to the*

result from Fig 2? In practice, how would one estimate K from experimental measurements that are obtained at a few time points? This seems to be an important explanation that is missing. Further, how could one assess the uncertainty associated with the estimate of K? The experimental measurements also appear to be measurements of the average concentration in the media or the spheroid. Presumably, this would impact estimates of K when the diffusive processes are not close to equilibrium?

Response: Thank you for your comment. To determine the amplification factor, we require the ratio of the effective diffusion coefficient inside the 3D cellular aggregate (3DCA) to the diffusion coefficient in the free medium, as indicated by equations (2) and (5). This ratio is a function of the porosity (ϵ) and tortuosity (τ) of the 3DCA, which represent its geometrical properties in terms of cell numbers and volumes. However, if these geometric parameters are unavailable, one can utilize the PBS with a range of effective diffusion coefficients (D_{eff}) alongside the proposed experimental approach to estimate D_{eff} (and thus, the corresponding k value).

We note that the k value is not calculated based on the proposed experiments. The reviewer's ambiguity arises from our calculation of the k values when explaining PBS, which might suggest that we utilize these values in the simulator. However, it is crucial to clarify that the simulator operates independently of the k value. It only relies on diffusion coefficients and random molecular steps, enabling us to evaluate the k value as obtained in Proposition 1. To prevent any confusion, we have removed the k value calculations when explaining PBS.

Comment 6: *K is referred to as the amplification factor and experiments are performed transferring 3D cellular aggregates from a low glucose concentration to a high glucose concentration. What would happen if 3D cellular aggregates from a high glucose concentration to a low glucose concentrations? Is the term "amplification factor" still appropriate in this setting?*

Response: The amplification factor depends on the diffusion coefficient outside and effective diffusion coefficient within the 3DCA. Since the effective diffusion coefficient within the 3DCA is smaller than that in the free media outside, we anticipate a value of higher than 1. Consequently, the concentration at the inner boundary represents an amplified version of the concentration outside, as illustrated by equation (4). Importantly, the amplification factor is independent of the initial

concentrations of molecules inside (related to pre-culture) and outside (culture media). In such a scenario, the diffusion equations share the same amplification boundary conditions but distinct initial conditions are applied.

Conversely, in instances where the diffusion coefficient outside the 3DCA is lower than inside (e.g., when cells are surrounded by a less porous cellular structure), the k value would be lower than 1. Consequently, instead of amplification, we observe attenuation from the outside to the inside, while amplification manifests from the inside to the outside.

We have highlighted this point after presenting Proposition 1 by the following sentence:

"In the general scenario involving the interface between two arbitrary diffusion environments, it is possible to encounter a situation where $k < 1$, which is inconsistent with the concept of an *amplification* factor. We employ the term "amplification" to emphasize the elevated concentration observed within a 3DCA."

Comment 7: *Experimental methods - How is the glucose concentration measured in the spheroids? The methods appear to focus on how to measure the glucose concentration in the media.*

Response: Thank you for your comment. As the reviewer mentions, the measurement of glucose is done in the medium. The glucose concentration could potentially be measured in the cells but this would be an endpoint assay, i.e. the cells have to be lysed to release the glucose, hence it would require more cellular material. By measuring the glucose in the medium we can follow the glucose concentration over time from the same sample.

"We note that the glucose concentration could potentially be measured in the cells but this would be an endpoint assay, i.e. the cells have to be lysed to release the glucose, hence it would require more cellular material. However, the experiments provided measure the concentration in the culture media surrounding the spheroids to infer the amplification factor. "

Comment 8: *Results focus on early time measurements over the course of 10 minutes. Due to this short timescale are the authors able to provide more details on how the 3D cellular aggregates are transferred between the different glucose conditions. Does this impact the results?*

Response: Thank you for your comment. The change of glucose concentration is not done by

transferring the spheroids but by changing the medium surrounding the spheroids. The spheroids are pre-cultured in medium with 2.80 mM glucose in a larger well with a larger medium volume to try to wash out as much glucose as possible from the spheroids. The spheroids are then transferred to a smaller well in order to perform the glucose measurements. The smaller well is chosen to be able to incubate in a smaller volume (75 or 100 μL). After transfer to the smaller well all medium is removed from the spheroids and the HepaRG medium with 11 mM glucose is added. There is a very small volume medium retained at the medium removal that is anticipated to have a minor contribution to the 75 and 100 µL incubation volume and hence negligible effect on the results. After the change of medium, aliquots are taken at 1, 5 and 10 minutes and glucose concentration is measured in the aliquots.

Minor Comments:

Comment 9: *Line 87 – typo – "insider"*

Response: Thank you. It is corrected in the revised manuscript.

Comment 10: *Line 206-209 – it would be helpful to mention your previous study in the introduction.*

Response: Thank you. In the revised manuscript, we have cited our previous study ([21]) in the introduction.

Comment 11: *Line 258-259 – "Initially the molecule concentration in the spheroids is expected to be $Cs < kCp$" – Could you add further justification for why this is the case. This also appears on Line 276.*

Response: Thank you for your comment. We have corrected this in the revised manuscript by changing the inequality to equality. As we have considered simplifying assumptions such as no reaction and equilibrium, we do not need to bound C_s. We have accordingly revised the relevant equations. It is important to emphasize that this adjustment does not affect our results or discussions.

Comment 12: *Line 280 – Does equilibrium ever occur in this system?*

Response: Thank you for your comment. The equilibrium assumption in the subsection "Rapid concentration reduction in culture medium" facilitates an intuitive explanation of how the revealed boundary conditions affect the concentration difference of molecules inside and outside the 3D cellular aggregate (3DCA). This section also offers calculations to aid experiment design and clarify the phenomenon of rapid concentration reduction at the outset. Notably, these equations have not been utilized in our results.

Comment 13: *Line 300-301 – Please clarify the calculation used to obtain* $1.7x10^{-}15$.

Response: Thank you for your comment. We have clarified this by revising the related sentence as follows:

"The diameter of differentiated HepaRG cells has been reported as 17 μm [26]. The volume of HepaRG cells is calculated to be approximately 1.7×10^{-15} m^3, as a mean reference of volumes assuming a spherical or cubic shape with this diameter, 2.5×10^{-15} m^3 and 0.9×10^{-15} m^3, respectively."

Comment 14: *Line 353 – Please add extra justification for the scaling.*

Response: Thank you for your constructive comment. In the revised manuscript, we have added the following sentence to better clarify the justification:

"For the step inside the spheroid, the molecule diffuses at a slower pace, represented by a reduced diffusion coefficient in the model, which diminishes the size of the step by the square root of the ratio of diffusion coefficients."

Comment 15: *Line 376 – Where did the variance of 0.15 come from? Does this agree with the experimental data?*

Response: Yes, it is the variance observed from the experimental data.

Comment 16: *Line 395 – What is meant by "pre-culture"?*

Response: Thank you for your comment. In this case, pre-culture refers to how the stellate cells (HHSteCs) were cultured (treated) before the formation of the spheroids.

Comment 17: *Line 419 – What is meant by "standard"?*

Response: Thank you for your comment. The standard refers to the medium that the HepaRG cells are normally cultured in and what is often recommended by suppliers. This medium can be altered for experimental purposes, e.g., here we have used lower glucose concentration during parts of the culture.

Comment 18: *Line 482 – "marginally significant" – The phrasing of these results in the conclusion on line 499-500 seems more appropriate.*

Response: Thank you for your comment. In the revised manuscript, the sentence has been revised as follows:

> "These values indicate a significant difference for both hypotheses."

Comment 19: *Fig 1 caption - is D_{diff} the same as D_{eff}?*

Response: Thank you for your accurate comment. Yes, it is corrected in the revised manuscript.

Comment 20: *I am not sure that the author summary is required for PLoS One from the submission guidelines.*

Response: Thank you for your comment. We have removed it in the revised manuscript.

Comment 21: *Code availability – Is the code going to be made available?*

Response: We have added the code as an Appendix in the revised manuscript.

Response to Reviewer 4

Overal Comment *This manuscript by Arjmandi et al presents a mixed computational/experimental investigation of glucose uptake in a 3D cell aggregate (3DCA) as an example of the more general mass transport case of nutrient transport within a 3DCA. The mathematical proof demonstrates that there is a locally increased concentration of glucose at the outer periphery of the 3DCA that corresponds to the mismatch between diffusion gradients in free media versus the 3DCA. This is an interesting and under-appreciated phenomenon in 3DCA-relevent studies, and this manuscript provides additional and useful information to this field. In general, the mathematical and theoretical considerations presented within this manuscript are technically sound and well-described. The authors then validate predictions of glucose uptake of 3DCA from media using serial timed media collections with differing initial media volume. The presented experimental conditions are well-described and technically sound. Predictions are then shown to match experimental conditions, validating the theoretical model of glucose uptake. In general, the manuscript is technically sound, scientifically rigorous, well-described, and well-written. However, there are a few additional points of consideration that the authors should address that would improve interpretations drawn from the manuscript.*

Response: Thank you sincerely for your supportive perspective and invaluable comments. We believe that your comments have significantly enhanced the quality of our revised manuscript.

Comment 1 *There is extensive data supporting the amount of glucose taken up by the 3DCA by demonstrating loss of free glucose in the cell culture media. Timed-collection experiments show the decay in glucose that are attributable to transport within the 3DCA. These experiments validate predictions of the amount of glucose taken up by the 3DCA, but these studies do not validate the increased distribution of glucose concentration within the periphery of the 3DCA. As the major finding reported by this manuscript is the 'amplification factor' that characterizes this locally increased,*

it is curious that the authors did not perform any studies to validate this phenomenon. There are several experimental designs with labeled analogs of glucose (or dyes of similar molecular weight) that could have been utilized to validate this finding. The authors should either perform these types of validation studies or discuss this lack of validation to mitigate their conclusions.

Response Thank you for your comment. The amplification factor proposed by Proposition 1 describes the local concentration increase as a boundary condition (BC). However, it inevitably results in an overall increase in molecular concentration within the 3DCA. In other words, the number of molecules absorbed inside the 3DCA directly relates to this factor because it couples the diffusion equations inside and outside the 3DCA via the BC (see equations (3)-(4)). A higher amplification factor leads to a greater uptake of molecules by the 3DCA. Considering this phenomenon, we have designed the experiment in which the glucose concentration is measured in the culture media (and not just the peripheral concentration). Thus, our proposed experiment aligns with the approach mentioned by the reviewer (please refer to the subsection ”Liver Spheroid Formation and Glucose Assay”). We have also provided an intuitive explanation of the impact of the identified boundary condition on the concentration of molecules within and surrounding the 3DCAs in the subsection titled ”Rapid concentration reduction in culture medium.”

For more clarification in the revised manuscript, we have added the following sentence in the subsection “Modeling of Diffusion BCs at the Border of 3DCA”:

“It is important to highlight that while the amplification factor characterizes the BC at the border, it influences the whole diffusion process both inside and outside the 3DCA by coupling the diffusion equations of both sides through the corresponding BC. ”

Comment 2 *The experimental conditions reflect an interesting thought experiment, but realistic cell culture conditions do not commonly apply such drastic step changes in glucose concentrations. In this manuscript, 3DCA are cultured in low glucose media and then transferred to high glucose media upon initiation of the experimental conditions. This was likely done to accentuate the initial glucose uptake in the 3DCA. However, it is difficult to draw comparisons with this experimental condition to that of a more common experimental condition in which 3DCA are maintained at similar glucose concentrations. How would this more common glucose concentration scenario be*

reflected? Will there still be the same 'amplification factor' if there is not a steep mismatch in glucose concentration? The authors need to discuss the interpretation of this data in a more common experimental scenario.

Response Thank you for this interesting comment. As explained in the introduction, an initial offset in glucose concentration has been reported by Casas et al. (2022) [17] with the similar 3DCA experiments in this manuscript. In this manuscript, we leverage our proposed PBS to design a pilot experiment that could provide a mechanistic explanation to the initial offset due to the amplification factor.

The specific experiment provided in this manuscript aims to verify the diffusion signal amplification factor mathematically proven in Proposition 1. This property is intrinsic to the system and is in principle independent of the instantaneous molecule concentrations inside and outside. Using a lower glucose concentration for the spheroid pre-culture was deliberate, aiming to magnify the subsequent change in concentration within the culture medium upon the addition of the 3DCAs, thereby facilitating better observation and understanding of the phenomenon.

As demonstrated by Equation (8), a larger difference between the glucose concentrations in the pre-culture medium C_p and culture medium C_m corresponds to a more substantial change in glucose concentration of culture medium following the addition of spheroids $(C_m - C'_m)$. For more clarification in the revised manuscript, we have underscored this aspect in the explanation of the experiment, specifically in the subsection "Liver Spheroid Formation and Glucose Assay," by including the following sentence:

"Taking Equation (8) into account, a larger difference between the glucose concentrations of 2.80 mM in the pre-culture and 11 mM in the culture media is anticipated to result in a sharper change in glucose concentration within the culture media following the addition of 11 mM medium to the spheroids."

Comment 3 *While not mentioned in the manuscript, it would be assumed that the local increase in glucose concentration at the periphery of the 3DCA would eventually equilibrate at steady state. For practical implications and application to future studies, how long is this 'amplification' maintained and what does the radial profile in glucose concentration look like prior to and during this*

equilibration period? This practical information is important to understand for applying this work to future 3DCA studies. If the time scale of equilibration of glucose concentration is much shorter than the time scale of the biological 3DCA culture experiment-of-interest, then this 'amplification' can be safely ignored. But if not, the findings in this paper are very important to consider. The authors should discuss time scale to better reflect the practical implication.

Response Thank you for considering this point. The amplification factor characterizes the BC at the border. As mentioned in response to comment 1, it inevitably affects the whole diffusion process in both environments resulting in an overall increase in molecular concentration within the 3DCA. In other words, the whole spatiotemporal concentration profile for the molecules inside the 3DCA directly relates to this factor because it couples the diffusion equations inside and outside the 3DCA. For PBS, we assume that the diffusion transient duration is shorter than the time required for internal reactions, implying that diffusive equilibrium is reached before reactions take place. This suggests that the amount of glucose within the 3DCA can be significantly higher than if a unitary boundary condition is assumed. Even if the reaction that consumes the molecules is faster, the amplification factor impacts the number of molecules entering the spheroid, thereby increasing the quantity of molecules consumed.

Therefore, the amplification property remains constant at the border, whether during the transient or equilibrium phases of diffusion, regardless of whether diffusion or reaction is faster. It constitutes a foundational characteristic of the 3DCA, independent of temporal variations or internal reactions within the 3DCA.

In the subsection "Rapid concentration reduction in culture medium", we incorporate the simplifying assumption that molecule reactions with the cells are significantly slower than the diffusion leading to equilibrium. This is assumed to calculate the concentration change in the external media that is practically measurable. For clarification, we have included the following sentences in the subsection "Rapid concentration reduction in culture medium":

"We note that the simplifying assumptions in this subsection illustrate calculations where a concentration change outside the spheroid is readily measurable. We use these to design and justify our experiments and results. However, it is essential to emphasize that the amplification

factor remains constant and influences the diffusion process, regardless of whether reactions within the spheroid occur at faster or slower rates."

Minor Comment The font size in axes labels and legends in many of the figures is quite small and difficult to read.

Response Thank you for your comment. We have updated the axis labels and legends for these figures.
