# Supplementary material for: 3D cell aggregates amplify diffusion signals
Source: PLoS One. 2024 Sep 12;19(9):e0310109. doi: 10.1371/journal.pone.0310109 (PMC12139657; doi:10.1371/journal.pone.0310109)
Supplement: Attachment — Submitted filename: Response to Reviewers.pdf [file pone.0310109.s009.pdf]

Response to the Reviewer's Comments for the Manuscript: '3D Cell Aggregates Amplify Diffusion Signals'

We would like to thank the Editor and reviewers for their helpful comments and valuable suggestions for our manuscript. Also, we would like to thank the Editor for his timely processing of our manuscript. We also thank both reviewers for their positive attitude toward this manuscript. Below, we provide a detailed response to the last comment of Reviewer 3. In the revised manuscript, we have corrected only a textual error in (9) and (10).

Response to Reviewer 2

Overall Comment: *The current revised version (R1) of the manuscript addressed all issues I raised previously. I can see a more detailed description of the methodology and discussion, overall the text. Specifically:*

- *introduction is now more objective.*
- *data presented in Fig 4, 5 and 6 is now discussed*
- *minor issues were addressed*

My opinion remains favorable to publication since there is a contribution to the field of organs-on-chip, improving the quality of the system, which let it be more reliable to physiological conditions.

Response: We would like to express our gratitude for your helpful comments and valuable suggestions, which have significantly improved our manuscript.

Response to Reviewer 3

Overall Comment: *The authors have thoughtfully and systematically addressed the comments that I raised. I have one comment outstanding that should be addressed prior to publication. I look forward to seeing the impact of this work in the field.*

Response: We would like to express our gratitude for your helpful comments and valuable suggestions, which have significantly improved our manuscript. We have responded to your comment below.

Comment 1: *Follow up to the authors response to Comment 14 regarding the scalings in Eq (9) and (10). The authors response states that for the step inside the spheroid the molecule diffuses at a slower pace which diminishes the step by the square root of the diffusion coefficients. In Eq (9),* $D > D_{eff}$ *so* $\sqrt{D/D_{eff}} > 1$ *will increase the step inside the spheroid rather than decrease it. Should the scaling be* $\sqrt{D_{eff}/D}$ *instead here? Similarly, in Eq (10) should the scaling be* $\sqrt{D/D_{eff}}$ *rather than* $\sqrt{D_{eff}/D}$*? If I am missing something additional clarification in the manuscript would be helpful. How does this influence the results?*

Response: Thank you for pointing out this issue. We have corrected this textual error in both (9) and (10) in the revised manuscript by swapping D and D_{eff}. However, the displacement vectors have been correctly updated in the code provided in the supplementary material. As a result, this typo in the equations in the paper does not affect the results. In the code, we use D_2/D_1 for molecules in the medium hitting the spheroid boundaries and D_1/D_2 for molecules inside the spheroids hitting the spheroid boundaries. Here, D_1 and D_2 represent the diffusion coefficient in the medium (D) and the effective diffusion coefficient within the spheroid (D_{eff}), respectively.
